# Validation of a methylation-based, tissue-free MRD assay in colorectal cancer patients from the GALAXY study

**DOI:** 10.1038/s41698-026-01277-5

**Published:** 2026-01-19

**Authors:** Yoshiaki Nakamura, Johannes G. Reiter, Prashanthi Natarajan, Joshua Babiarz, Preethi Srinivasan, Jayashree Joshi, Yu Lin, Tzu-Chun Chen, Nathan Liang, Su Maw, Ehsan Haghshenas, Garima Kushwaha, Boris Gutman, Ilker Tunc, Wen-Ching Chan, Antony Tin, Yuefan Huang, Sara L. Bristow, Meenakshi Malhotra, Shruti Sharma, Stephanie A. Sanchez, Adham Jurdi, Minetta C. Liu, Trupti Kawli, Matthew Rabinowitz, Alexey Aleshin, Daisuke Kotani, Oki Eiji, Takayuki Yoshino

**Affiliations:** 1 Department of Gastroenterology and Gastrointestinal Oncology, National Cancer Center Hospital East, Kashiwa, Japan; 2 Natera, Inc, Austin, TX USA; 3 Department of Surgery and Science, Graduate School of Medical Sciences, Kyushu University, Fukuoka, Japan

**Keywords:** Biomarkers, Cancer, Gastroenterology, Oncology

## Abstract

This study validates a methylation-based, tissue-free assay (Latitude^TM^ assay) for detecting molecular residual disease (MRD) in colorectal cancer (CRC), analyzing data from 195 patients (1230 timepoints) from the observational GALAXY study. The assay demonstrated that ctDNA-positivity correlated with significantly worse disease-free survival (DFS) in both the MRD (HR = 10.0, *P* < 0.001) and post-definitive treatment surveillance windows (HR = 31.9, *P* < 0.001). In the MRD window, the assay demonstrated a sensitivity of 58.5% (38/65), while the specificity in patients who did not receive adjuvant chemotherapy (ACT) was 100% (63/63). Longitudinally, relapse was detected with a sensitivity of 84.4% (54/64), with high specificity at the patient (92.1%; 116/126) and sample (97.2%; 619/637) levels. Median lead time from first circulating tumor DNA (ctDNA)-positive result to relapse was 4.6 months. For high-risk stage II and III CRC, ctDNA-positive patients benefited from ACT (adj.HR = 0.014, *P* < 0.0001), unlike ctDNA-negative patients. These findings highlight the robust prognostic and predictive capabilities of this tissue-free MRD assay in CRC patients.

## Introduction

Globally, colorectal cancer (CRC) is the third most common diagnosis and the second most common cause of mortality among cancer patients^1^. From 2020 to 2040, the number of new CRC cases and deaths is expected to increase by 63% (1.93–3.2 million) and 73% (0.9–1.6 million), respectively^2^. While the standard of care in patients with stage II or III CRC is surgical resection with the option of subsequent adjuvant chemotherapy (ACT) depending on clinicopathological risk factors^3,4^, more than 30% of patients will experience recurrence following resection^5,6^.

Personalized, tumor-informed circulating tumor DNA (ctDNA) has been established as a reliable prognostic and predictive biomarker for detecting post-surgical molecular residual disease (MRD) ahead of clinical recurrence, and for identifying patients who may benefit from ACT^7,8^. Studies with a tumor-informed approach have shown a strong alignment and preservation of ctDNA variants between tumor tissue and subsequent plasma samples, yielding high sensitivity and specificity^9,10^. While these personalized approaches are highly accurate, the need for tissue adds time and cost^11^. Tissue-free ctDNA tests are ideal when tumor samples are either unavailable due to biological or logistical reasons or when samples are of suboptimal quality for DNA extraction.

The GALAXY study is an observational arm of the CIRCULATE-Japan trial that analyzed presurgical and postsurgical ctDNA using a tumor-informed assay in patients with clinical stage II-IV or recurrent CRC after curative intent surgery^7,8^. The study showed postsurgical ctDNA positivity to be associated with a higher risk of recurrence and benefit from ACT. Here, we present the clinical validation of a methylation-based tissue-free MRD assay (Latitude^TM^ assay) on a subset of patients from the GALAXY study. In this analysis, we report on the post-surgical ctDNA positivity rate and its association with disease-free survival (DFS) during the post-surgical MRD window and also assess its clinical performance and utility in a longitudinal setting.

## Results

### Patient characteristics

**Fig. 1:**
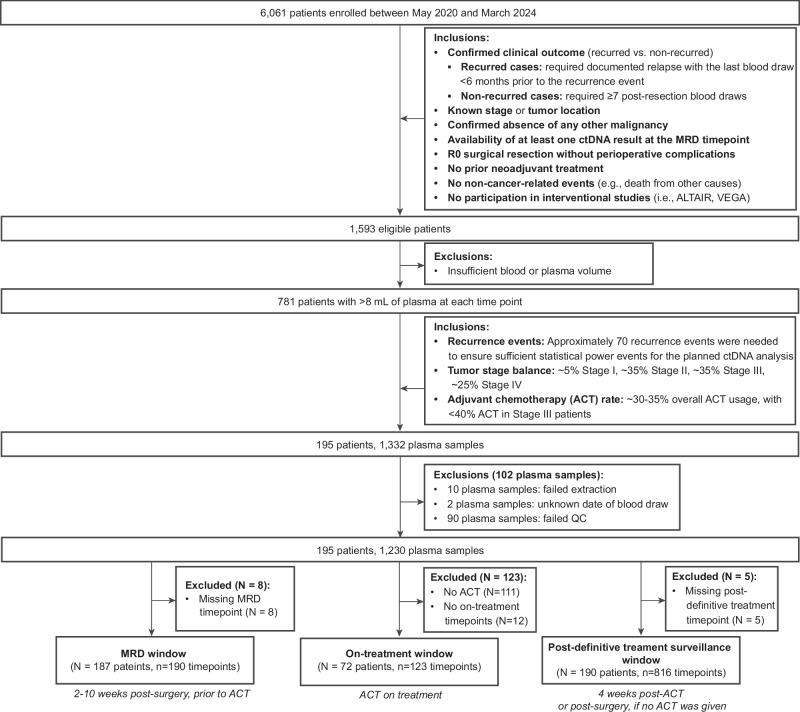
Patient selection. CONSORT diagram illustrating the inclusion of patients from enrollment to primary and subanalyses in this study.

Of 6061 patients with surgically resectable CRC enrolled in GALAXY between 8 May 2020 and 31 March 2024, 781 patients met the eligibility criteria with at least >8 mL of plasma sample available at each time point. Of these, 195 patients (pathological stages I-IV CRC) with 1230 plasma samples were selected, as pre-defined in the statistical analysis plan to meet the requirements of the performance analysis (Fig. 1). The median age of the cohort was 69 years (29–88 years), and the median follow-up was 28 months (range: 2–49 months). Patient characteristics at baseline are summarized in Supplementary Table 1. The complete clinical course of the patients, stratified by stage, including treatment and longitudinal ctDNA time points is presented in Supplementary Fig. 1.

### Association of ctDNA status in the MRD window with DFS

**Fig. 2:**
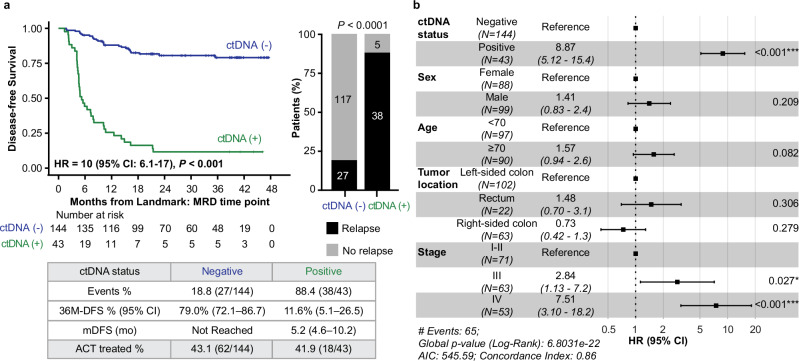
ctDNA status by a tissue-free assay during the MRD window is predictive of DFS in post-surgical patients with CRC. **a** Kaplan–Meier analysis of DFS and plasma ctDNA status during the MRD window. The bar plot shows the association between ctDNA status during the MRD window and relapse during the study period. **b** Forest plot depicting the multivariate analysis (including ctDNA status during the MRD window and clinicopathological features) for DFS. Various prognostic factors and their association with DFS, as indicated by HR, were analyzed across the cohort using the two-sided Wald chi-square test. The unadjusted HRs (black squares) and 95% CIs (horizontal lines) are shown for each prognostic factor. The vertical line is the null hypothesis.

Of 195 patients who met the study inclusion criteria, 187 had ctDNA results available (190 plasma samples, median 1 (range: 1–2) ctDNA test per patient) within the MRD window (2–10 weeks post-surgery and before the start of ACT). Of these, 23.0% (43/187) were ctDNA-positive, of whom 88.4% (38/43) experienced recurrence, whereas only 18.8% (27/144) of ctDNA-negative patients experienced recurrence (HR: 10.0, 95% CI: 6.1–17.0, *P* < 0.001; Fig. 2a), demonstrating a 36-month DFS (from the landmark timepoint) of 11.6% (95% CI: 5.1–26.5%) for ctDNA-positive versus 79.0% (95% CI: 72.1–86.7%) for ctDNA-negative patients. Among all recurrences, 58.5% (38/65) were ctDNA-positive. Among those not detected in the MRD window, 66.7% (18/27) had a positive result at subsequent time points. Notably, all 5 patients with positive ctDNA results who did not relapse received ACT. Among the non-relapsed patients who did not receive ACT (*N* = 63), specificity was 100%. To further evaluate the prognostic value of ctDNA status compared to other clinicopathological risk factors, we performed a multivariate analysis. ctDNA positivity was observed to be the most significant prognostic factor associated with inferior DFS (HR: 8.87, 95% CI: 5.12–15.4, *P* < 0.001) (Fig. 2b), followed by increasing pathological stage (stage III: *P* = 0.027, stage IV: *P* < 0.001).

### Association of ctDNA status in the post-definitive treatment surveillance window with DFS

**Fig. 3:**
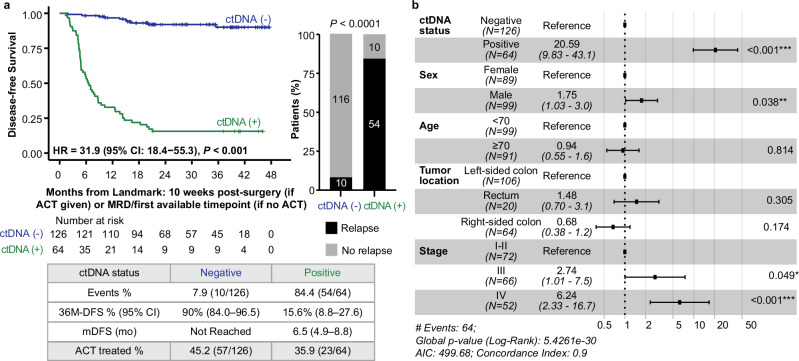
ctDNA status in the post-definitive treatment surveillance window is predictive of DFS in post-surgical patients with CRC. **a** Kaplan–Meier analysis of DFS and plasma ctDNA status during the post-definitive treatment surveillance window. The bar plot shows the association between ctDNA status during the post-definitive treatment surveillance window and relapse during the study period. **b** Forest plot depicting the multivariate analysis (including ctDNA status during the post-definitive treatment surveillance window and clinicopathological features) for DFS. Various prognostic factors and their association with DFS, as indicated by HR, were analyzed across the cohort using the two-sided Wald chi-square test. The unadjusted HRs (black squares) and 95% CIs (horizontal lines) are shown for each prognostic factor. The vertical line is the null hypothesis.

During the post-definitive treatment surveillance window (4-weeks post-ACT or post-surgery if no ACT was given), ctDNA status was available for 190 patients (816 plasma samples; median 4 (range: 1–8) test per patient); ctDNA-positivity was observed in 33.7% (64/190) of patients, of whom 84.4% (54/64) experienced recurrence, whereas only 7.9% (10/126) of patients with serial ctDNA-negative status experienced recurrence (HR: 31.9, 95% CI: 18.4–55.3, *P* < 0.001; Fig. 3a), demonstrating a 36-month DFS (from the landmark timepoint) of 15.6% (95% CI: 8.8-27.6%) for ctDNA-positive versus 90.0% (95% CI: 84.0–96.5%) for ctDNA-negative patients. ctDNA-positivity demonstrated a patient-level sensitivity and specificity of 84.4% (54/64) and 92.1% (116/126), respectively, and a sample-level specificity of 97.2% (619/637). In the multivariate analysis, ctDNA-positivity during post-definitive treatment surveillance was the strongest prognostic factor associated with poor DFS (HR: 20.59, 95% CI: 9.83–43.1, *P* < 0.001), followed by stage IV disease (*P* < 0.001) and male (*P* = 0.038) (Fig. 3b). Among colon cancer patients, patient-level sensitivity and specificity were 87.0% (47/54) and 91.4% (106/116), respectively.

### Association of ACT and MRD status with DFS, and the impact of on-treatment ctDNA dynamics

**Fig. 4:**
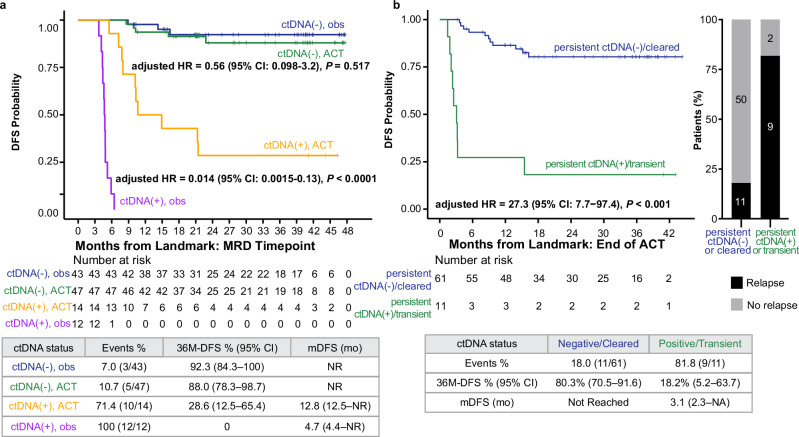
ctDNA testing is predictive of the benefit of ACT in postsurgical patients with colorectal cancer. **a** Kaplan–Meier analysis of DFS stratified by adjuvant treatment (observation versus ACT) in ctDNA-positive and -negative patients with pathological high-risk stage II or stage III colorectal cancer. **b** Kaplan-Meier analysis of DFS and plasma ctDNA status in all patients who underwent ACT. The barplot shows the association between ctDNA status during ACT and relapse during the study period.

Since ACT treatment is recommended for patients with high-risk stage II disease and all patients with stage III disease, we further stratified the analysis by ACT status in this subgroup (*N* = 116). To decrease the chance of disease and patient-related bias, we adjusted for confounding variables such as age, sex, and tumor location in this analysis. Of the patients in this subgroup, 22.4% (26/116) were ctDNA-positive; of these, 53.8% (*N* = 14/26) received ACT, whereas 77.6% (90/116) were ctDNA-negative, and 52.2% (47/90) of whom received ACT. We observed that ctDNA-positive patients derived benefit from ACT (adj HR: 0.014, 95% CI: 0.0015–0.13, *P* < 0.0001; Fig. 4). Conversely, no statistically significant benefit from ACT was observed for ctDNA-negative patients (adj HR: 0.56, 95% CI: 0.098–3.2, *P* = 0.517). The p-interaction test comparing the effect of ACT treatment by ctDNA status was statistically significant (HR: 0.010, 95% CI: 0.00078–0.13, *P* = 0.0004).

We further assessed the impact of ACT on ctDNA status with DFS among all patients who received ACT (N = 72). A median of 2 (range: 1–3) ctDNA tests were performed per patient in the adjuvant setting. Patients were categorized into a ctDNA-negative group (*N* = 61) if they remained negative or achieved clearance on ACT or ctDNA-positive (*N* = 11) if they experienced transient clearance or remained consistently ctDNA-positive. The ctDNA-positive group had a significantly higher recurrence rate of 81.8% (9/11) compared to 18.0% (11/61) in the ctDNA-negative group. This ctDNA-positivity during ACT was highly prognostic of an inferior DFS (adjHR: 27.30, 95% CI: 7.65–97.35, *P* < 0.001; Fig. 4b), with a 36-month DFS of only 18.2% (95% CI: 5.2–63.7%) for ctDNA-positive patients versus 80.3% (95% CI: 70.5–91.6%) for ctDNA-negative patients.

### Association of any time ctDNA positivity with sites of recurrence and lead time analysis

**Fig. 5:**
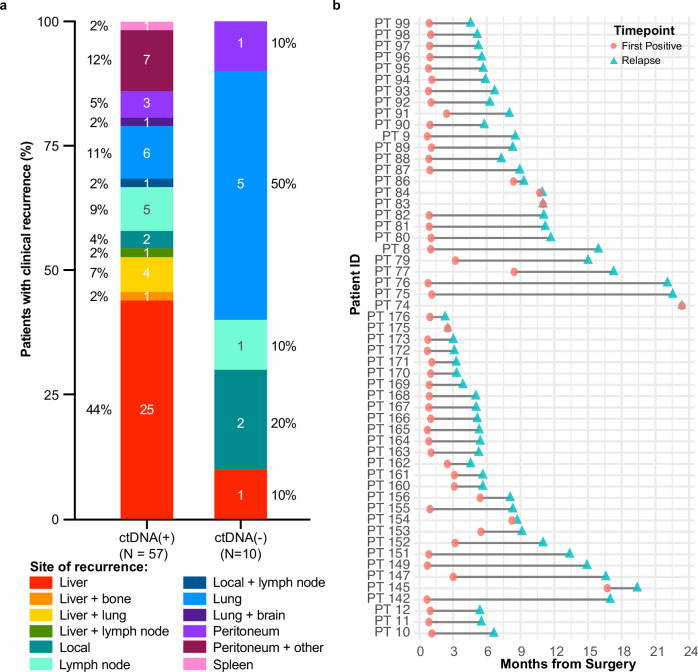
Any time ctDNA results. **a** Bar plot showing the association of the percentage of patients with ctDNA positivity (*N* = 57) and negativity (*N* = 10) at any time after surgery with the site of recurrence. The number of patients is reported in the corresponding bar chart and the ctDNA positivity rate at each metastatic site is reported in percentage adjascent to the bar chart. **b** Line chart depicting the time from the first ctDNA positive result to clinical relapse for 55 patients.

We next assessed the association between ctDNA status (positive or negative at any time) and recurrence site. Of the 68 patients who experienced recurrence, 67 had at least one ctDNA result, with the exception of one patient who had no ctDNA result prior to relapse (brain and lung). Among patients with post-surgical ctDNA positivity (*N* = 57), liver metastases were most common (43.9%, 25/57), followed by peritoneum+other (12.3%, 7/57), lung (10.5%, 6/57). The median time to recurrence (TTR) from surgery for ctDNA-positive patients with liver metastases was 6.6 months, compared to 8.2 months for other metastases (*P* = 0.33). For ctDNA-negative patients, lung was the most frequent recurrence site (50.0%, 5/10), with a median TTR of 16.13 months (range: 2.8–22.3 months) (Fig. 5a).

Among patients with any time ctDNA-positivity prior to recurrence (*N* = 55), the median lead time from the first positive ctDNA result to documented relapse was 4.6 months (range: 0–21.5 months). The median TTR in these patients was 6.7 months (range: 2.3–23.4 months) from surgery (Fig. 5b).

## Discussion

The tissue-free MRD assay utilized in this study detects ctDNA by targeting a panel of cancer-specific DNA methylation markers using next-generation sequencing technology. Since DNA methylation changes can occur early in cancer development^12^, methylation-based assays provide a robust and standardized approach for detecting ctDNA, even at low concentrations, which is beneficial for identifying cancer either early or in early stages^13–15^. Although tumor-informed ctDNA assays that utilize somatic mutations are known to provide a more sensitive MRD detection method with greater confidence in identifying low-frequency mutations and reducing the false-positive rate caused by biological nonspecificity^15,16^, the advantage of using ctDNA methylation markers is appealing in cases where tumor tissue is unavailable or inaccessible^17^. Moreover, methylation patterns can also be used to monitor treatment response and detect early signs of resistance, allowing personalized treatment strategies based on epigenetic changes^18^.

The findings from our study have validated the clinical performance of a tissue-free, methylation-based MRD assay (Latitude assay) in patients with stage I–IV CRC. Our data, consistent with prior research with ctDNA assays, highlights the prognostic value of post-surgical ctDNA at a single time point (i.e., MRD window), demonstrating a sensitivity of 58% (38/65), and a specificity in patients who did not receive ACT of 100% (63/63). Moreover, the integration of longitudinal testing during the post-definitive treatment surveillance window enhanced sensitivity to 84.4% (54/64), while preserving high specificity at both the patient (92.1%; 116/126) and sample (97.2%; 619/637) levels. Furthermore, the ctDNA-positivity by the tissue-free MRD assay preceded radiological relapse by a median of 4.6 months (range: 0–21.5 months). Overall, ctDNA-positivity using the tissue-free MRD assay emerged as a significant prognostic factor associated with worse DFS at both the MRD (HR: 10, *P* < 0.001) and post-definitive treatment surveillance (HR: 31.9, *P* < 0.001) windows. These findings are comparable with results previously reported for a personalized, tumor-informed ctDNA assay (Signatera^TM^), where the prognostic ability of the assay for detecting recurrence was significant in the MRD (HR: 11.99, *P* < 0.0001) and surveillance windows (HR: 33.56, *P* < 0.0001) in the full GALAXY cohort^8^.

In addition, our data demonstrates that ctDNA-positivity during the MRD window is predictive of the benefit of ACT (adj. HR: 0.014, *P* < 0.0001), while patients with ctDNA-negativity showed no significant difference in outcomes between patients who received ACT and who did not receive ACT (*P* = 0.51). Furthermore, the effect of adjuvant therapy differs significantly by MRD status (*P* = 0.0004). The hazard ratio of 0.01 for the interaction *P* value suggests a 99% reduction associated with adjuvant therapy in the MRD-positive patients compared to MRD-negative patients. Together, our results indicate that methylated ctDNA can prompt the presence of MRD, identifying patients at a high risk of relapse, and can help clinicians determine a subset of the population that would benefit most from treatment.

This assay was built upon a distinct, multipronged development strategy to ensure clinical robustness, as observed from the findings in this study. First, a comprehensive methylation biomarker discovery program was undertaken in samples across various clinicopathologic features, including CRC subtypes, histologies, stages, patient age, and self-reported race and ancestry, such that the assay’s panel not only includes high performing biomarkers but also is broadly applicable and clinically relevant across a heterogeneous population. Second, the assay workflow was designed to maximize the molecular recovery, which is critical for maximizing sensitivity, particularly at low ctDNA fractions typically encountered in the MRD setting. Finally, a proprietary and robust machine learning algorithm was developed for sample classification, which underwent extensive validation and testing across numerous independent cohorts. The efficacy and success of this comprehensive approach is demonstrated by the strong clinical performance and prognostic utility reported here.

Several studies have been published that incorporate the ctDNA-methylation assay for risk stratification in CRC. A recent study developed a prognostic model using a five-marker cfDNA methylation panel in CRC patients, which outperformed other markers like serum CEA, TNM stage, or tumor location^19^. Pedersen et al. established a plasma BCAT1/IKZF1 methylation reference, improving specificity^20^, while Al Naji et al. found that combining BCAT1/IKZF1 ctDNA methylation with clinical factors enhances prediction accuracy^21^. Another recent prospective clinical trial (NCT00958737) studied the association of postoperative ctDNA methylation markers (WIF1 and NPY; as measured by digital droplet PCR) with tumor recurrence in stage II and III CRC patients. Results indicated a 2-year DFS of 82% for ctDNA-negative patients vs. 64% for ctDNA-positive, suggesting the ability of ctDNA methylation analysis to predict MRD^22^. The authors further evaluated these methylation markers in a randomized trial (PRODIGE-GERCOR IDEA-France) with stage III patients, investigating the benefit of 3 vs 6 months of ACT^23^. Post-surgery ctDNA was found to be an independent prognostic marker for DFS and OS, especially in T4 tumors, N2 disease, or those treated for 3 months. However, the study noted a lower ctDNA positivity rate (13.8%) compared to NGS-based studies, with reduced sensitivity and predictive value—5-year DFS was 65% for ctDNA-positive versus 73% negative^17^. An interim readout of the FIND trial (NCT05904665), a multicenter, prospective, phase III, randomized controlled cohort study, demonstrated the utility of ctDNA-based surveillance in non-metastatic CRC patients being monitored with a single-tube methylation-specific quantitative PCR (mqMSP) assay. This assay detects 10 methylation markers and analyzes plasma samples with tumor DNA as low as 0.05%. The results indicated that ctDNA-guided surveillance doubled the proportion of recurrent patients receiving curative-intent treatment and detected recurrent lesions 3.05 months earlier than standard imaging^24^. Recently, a study of a tissue-free epigenomic MRD assay that utilized methylation-based markers demonstrated ctDNA to be prognostic of the recurrence-free interval for stage I-III CRC patients during the MRD (HR: 6.48, *P* < 0.0001) and surveillance (HR: 16.70, *P* < 0.0001) settings^25^. An evaluation of another tumor-naive ctDNA assay found ctDNA to be associated with DFS in both the MRD (SNV and methylation markers, HR: 7.28) and surveillance (methylation markers only, HR: 21.9)^26^. These results are promising and further reinforce the utility of methylation-based ctDNA assays.

Our study presents prospective ctDNA analysis in a sub cohort of patients with long-term follow-up, but our study is associated with some limitations. To ensure statistical rigor and appropriate confidence interval widths for performance estimates, the cohort was selectively refined to include ~70 recurrence events and to mirror the stage distribution and ACT usage of the broader GALAXY study population. Although this approach allows internal validity, it might restrict the generalizability of the cohort to real-world populations, which are prone to variability in treatment patterns and disease features. However, to account for potential biases in patient characteristics, we performed multivariate analyses, demonstrating the clear prognostic nature of ctDNA in both the MRD and the post-definitive treatment surveillance windows. Furthermore, to account for immortal time bias, all analyses were landmarked and in the post-definitive treatment surveillance window, to account for multiple ctDNA timepoints interrogated at different intervals, a time variate co-variate methodology was implemented to estimate the hazard ratio. The ability to perform the Latitude test provides clinicians with a unique opportunity to order an NGS-based ctDNA-assay in clinical cases when sufficient tumor tissue is unavailable or a quick answer is critical for patient management. Additional prospective trials and real-world studies utilizing the tissue-free MRD assay would further validate the clinical utility of the assay and are currently underway.

In summary, we report on the clinical performance of a tissue-free, methylation-based MRD assay for ctDNA detection in patients with stage I-IV CRC. We showed that ctDNA positivity was strongly associated with DFS and predictive of ACT benefit, demonstrating robust performance across both the MRD and the post-definitive treatment surveillance windows. The implementation of prospective randomized trials will further investigate the optimal ctDNA-guided treatment strategy for surgically resectable CRC.

## Methods

### Cohort characteristics and selection

To assess the performance of the methylation-based tissue-free MRD assay (Latitude assay), a subset of patients from the GALAXY study was used. GALAXY is the observational arm of the ongoing prospective, multicenter CIRCULATE-Japan platform trial (UMIN000039205). The study serves to screen patients with ctDNA-based MRD status, which determines their assignment to either the ALTAIR (treatment escalation) or VEGA (treatment de-escalation) randomized phase III, ctDNA-guided interventional trials. Patients enrolled in GALAXY who had stored plasma of sufficient volume (de-identified residual patient samples) and 2-year clinical follow-up were eligible for inclusion in this study. Per protocol, blood samples were serially collected at weeks 4 (MRD time point), 12, 24, 36, 48, 72, and 96 post-surgery. Additional blood samples were collected and banked under the GALAXY protocol. A retrospective analysis was subsequently performed using these banked specimens.

A representative cohort was selected to reflect the stage and treatment distribution based on historical cohorts and previous readouts from the observational GALAXY study (Supplementary Table 1). The key inclusion criteria for cohort selection were: availability of confirmed clinical outcome (recurred vs. non-recurred), known stage or tumor location, confirmed absence of any other malignancy, availability of at least one ctDNA timepoint within the MRD window, R0 surgical resection without perioperative complications, no history of prior neoadjuvant treatment, no non-cancer-related event (i.e., death from any cause), and no participation in other interventional studies. Additional requirements for the recurred cases included a documented relapse with the last blood draw <6 months prior to the recurrence event. The requirement for the non-recurred cases included a total of ≥7 post-resection blood draws to ensure consistent longitudinal follow-up, unaffected by early withdrawal, adverse events, or non-compliance. This requirement was not applicable to recurrent patients, as ctDNA monitoring in GALAXY was discontinued at the time of relapse. The exclusion criteria included patients with insufficient blood or plasma volume for ctDNA analysis. Samples that failed quality control metrics/extraction were also excluded.

In order to estimate the sensitivity and specificity of the tissue-free MRD assay in predicting recurrence, the cohort was enriched for patients with recurrent disease and balanced to have a stage distribution similar to the GALAXY study, representing ~5% stage I, ~35% stage II, ~35% stage III, ~25% stage IV. Furthermore, the cohort was balanced to include an overall ACT usage of ~30–35%, with <40% ACT usage in stage III patients and other critical clinical and pathological factors including, sex, ECOG score, pathological N stage, ACT usage, *BRAF* V600E, *RAS* mutation status, microsatellite status as summarized in Supplementary Table 1. Given the aim to make the analyzed subset representative of the broader GALAXY cohort, no significant differences were observed between the analyzed subset and the broader GALAXY cohort for these variables. This selection enabled an estimate of sensitivity and specificity with a margin of error of no more than +/-12 percentage points.

### Methylation-based, tissue-free MRD assay (Latitude assay)

#### Plasma DNA library and next generation sequencing

Blood samples were collected in two 10 mL cfDNA blood collection tubes. A median of 10.2 mL plasma (range: 2.4–10.2 mL) was isolated for cfDNA extraction. Up to 100 ng (33,000 haploid genome equivalents) of cfDNA was then used from each plasma sample as input into library preparation. The cfDNA undergoes end repair, ligation, and amplification for sequencing-based detection of methylated cytosines. The resulting libraries are enriched for CRC-associated differentially methylated regions (DMRs) and sequenced on an Illumina NovaSeq X. Reads are subsequently mapped to the hg19 reference genome and deduplicated.

#### Sequencing pipeline

The raw methylation sequencing data generated from the NGS is processed through a dry lab pipeline. This process involves demultiplexing the sequencing reads from multiple samples, followed by adapter removal via a trimming step. The reads are then aligned to the genome, and the status of each cytosine is annotated as methylated or unmethylated. Subsequently, PCR duplicates are removed. Finally, sequencing reads corresponding to the targets of interest are extracted and passed on to the sample classification pipeline, while reads for control regions are passed on to the corresponding pipelines for tracer and contamination analyses.

#### Sample classification pipeline

Sequenced data that passes the quality control is processed through a sample classification pipeline that incorporates a machine learning model. This model was previously trained on a separate cohort of CRC samples compared to negative controls to maximize classification performance and achieve a desired analytical specificity of 99%. For each target, the differentially methylated fragments rate is calculated as the fraction of these fragments per genomic target region. The hypermethylated fragment rates are then fed into a machine learning model, which predicts whether the sample is ctDNA-positive or ctDNA-negative.

#### Pipeline QC controls

Analysis of cfDNA includes batch-level run controls, and sample-level QC metrics to ensure run and sample analysis integrity. Spike-ins are included in each sample to ensure protection and conversion efficiency. Each sample batch contains a well-characterized, unmethylated reference cell line as a ctDNA-negative sample and a mixture of in vitro methylated DNA in a background of unmethylated DNA as a ctDNA-positive sample. A no-template control is included to assess run-to-run or carryover contamination.

### Statistical analysis

The MRD window was defined as 2–10 weeks post-surgery and prior to the initiation of any adjuvant therapy. The post-definitive treatment surveillance window spanned from the MRD window until the last follow-up or clinical recurrence if no ACT was given or 4 weeks after ACT until the last follow-up or clinical recurrence if ACT was given. The primary outcome was DFS, defined as the time from the landmark timepoint to recurrence. Recurrence was determined by diagnostic imaging or any other diagnostic procedure if imaging was not confirmatory (e.g., colonoscopy to diagnose a local recurrence). Living patients without a documented event were censored on the date of final survival confirmation. The Kaplan-Meier method was used for estimating survival distributions. Hazard ratios (HRs), associated 95% confidence intervals (CI), and *p* values were calculated using Cox proportional hazards regression. The log-rank test was used to compare survival distributions between two or more groups. To account for potential immortal time bias, MRD analyses were landmarked at the date of the MRD timepoint, and post-definitive treatment surveillance analyses were landmarked at the MRD timepoint (if no ACT was given) or 10 weeks after surgery (if ACT was given). To further account for potential immortal time bias, a time variate co-variate analysis was completed in the post-definitive treatment surveillance window. Additionally, Cox regression was used to compare the survival benefit between the ACT and observation groups and was landmarked at the MRD timepoint. Interaction effects between ctDNA status and ACT status were determined using multiplicative models. The association between ACT and ctDNA status with DFS in patients who received ACT was assessed by Cox regression, landmarked at the end of ACT, after all on-treatment ctDNA measurements had been completed. Both of these analyses were adjusted for age, sex, and tumor location. Survival analyses were conducted using R software v4.3.1, with the survival (version 3.8.3), survminer (version 0.5.0) packages. A multivariable Cox proportional hazards model was used to assess the most significant prognostic factor associated with DFS. All *p* values were based on two-sided tests, and differences were considered statistically significant at *p* ≤ 0.05.

### Ethics statement

All patients provided written informed consent before participation in the study. The analysis was conducted following approval by the institutional review board (IRB) of the National Cancer Center, Japan, and Natera’s IRB-approved protocol (Salus #24401) and adhered to the principles of the Declaration of Helsinki and ICH guidelines for Good Clinical Practices. institution. The GALAXY study is registered in the Japan Registry of Clinical Trials (UMIN000039205). The study was conducted in accordance with the Declaration of Helsinki.

## Supplementary information

Nakamura et al_Supplementary Material.

## Data Availability

The authors declare that all relevant, non-proprietary data used to conduct the analyses are available within the article. To protect the privacy and confidentiality of patients in this study under the Japanese Act on the Protection of Personal Information, identifiable clinical data are not made publicly available in a repository or in the supplementary material of the article but can be requested at any time from the corresponding author (T.Y. (tyoshino@east.ncc.go.jp) or E.O. (oki.eiji.857@m.kyushu-u.ac.jp)). Any requests will be reviewed within a timeframe of 2–3 weeks by the CIRCULATE-Japan study steering committee to verify whether the request is subject to any intellectual property or confidentiality obligations. All data shared will be de-identified and will be provided to researchers with access limited for scientific verification purposes and with strict prohibitions on secondary use.

## Code availability

The fully documented code and raw outputs for the R statistical computing environment for analyses related to this manuscript are deposited at the GitHub repository and can be accessed at https://github.com/Natera-TMED/tfMRD_CRC.
