# Supplementary material for: Validation of a methylation-based, tissue-free MRD assay in colorectal cancer patients from the GALAXY study
Source: NPJ Precis Oncol. 2026 Jan 19;10:70. doi: 10.1038/s41698-026-01277-5 (PMC12913904; doi:10.1038/s41698-026-01277-5)
Supplement: Supplementary file 1 — Nakamura et al_Supplementary Material. [file 41698_2026_1277_MOESM1_ESM.pdf]

Supplementary Material

Supplementary Table 1. Comparison of clinicopathological factors: Latitude clinical validation cohort vs. full Galaxy cohort.

Characteristic		*N* = 195	Nakamura 2024	Statistics
Age, median (range)	Years	68 (29-88)	69 (28-95)	p=0.7884
Sex, n (%)	Female	91 (47)	1091 (49)	p=0.602
	Male	104 (53)	1149 (51)	
Pathological stage, n (%)	I	7 (3.6)	234 (10)	p=0.001[a]
	II	66 (34)	652 (29)	p=0.165
	III	67 (34)	936 (42)	p=0.050
	IV	55 (28)	418 (19)	p=0.002[a]
Primary site, n (%)[b]	Right-sided colon	64 (33)	863 (39)	p=0.1243
	Left-sided colon	131 (67)	1377 (61)	
ECOG score, n (%)	0	171 (88)	2046 (91)	p=0.090
	1	24 (12)	194 (9)	
Pathological T stage, n (%)	T1-T2	12 (7.7)	317 (14)	p=0.002[a]
	T3-T4	151 (93)	1630 (73)	
	Unknown	32	293 (13)	
Pathological N stage, n (%)	N0	77 (47)	922 (41)	p=0.422
	N1-2	86 (53)	1025 (46)	
	Unknown	32	293 (13)	
ACT, n (%)	Adjuvant chemotherapy	84 (43)	946 (42)	p=0.821
	Observation	111 (57)	1294 (58)	
BRAF V600E	BRAF wildtype	183 (94)	2062 (92)	p=0.485
	BRAF V600E	12 (6.2)	178 (8)	
RAS, n (%)	Wildtype	124 (64)	1303 (58)	p=0.150
	Mutated	71 (36)	937 (42)	
MSI status, n (%)	MSS	180 (92)	2052 (90)	p=0.521
	MSI-High	15 (7.7)	215 (10)	
Radiological recurrence, n (%)	Yes	68 (35)	500 (22)	p=0.001[a]
	No	127 (65)	1740 (78)	
Recurrence site, n (% of 68 for this study, % of 500 for Nakamura et al 2024)	Liver	26 (38)	144 (29)	p=0.121
	Liver+lung	4 (6)	24 (5)	p=0.763
	liver+bone	1(1)	1 (<1)	p=0.225
	Liver+lymph node	1(1)	6 (1)	p=0.593
	Local	4 (6)	5 (1)	p=0.015[c]
	Local+lymph	1 (1)	1 (<1)	p=0.225
	Lung	11 (16)	128 (26)	p=0.0992
	Lung+brain	2 (3)	2 (<1)	p=0.0723
	Lymph node	6 (9)	29 (6)	p=0.292
	Peritoneum	4 (6)	56 (11)	p=0.212
	Peritoneum + other	7 (10)	63 (13)	p=0.697
	Spleen	1 (1)	1 (<1)	p=0.225
	Other	0 (0)	40 (8%)	p=0.009[c]

The latitude subset cohort was selected following the inclusion and exclusions as outlined in the consort diagram, including availability of sufficient blood volume (>8mL of plasma samples) to run Latitude assay. [a]The cohort was enriched for recurrent cases to ensure statistical power for the estimation of clinical performance, resulting increase in stage IV, and decrease in stage I and T1-T2 tumors. [b]Rectal patients (N=24) were combined with left-sided colon in “Primary site” to match the comparison withthe larger GALAXY study. cMinor variations in relapse sites are attributed to the sampling design and do not reflect a bias in tumor fraction.

Supplementary Figure 1

A) Swimmer Plot (Stage I-II): Timeline of Treatment and ctDNA Results

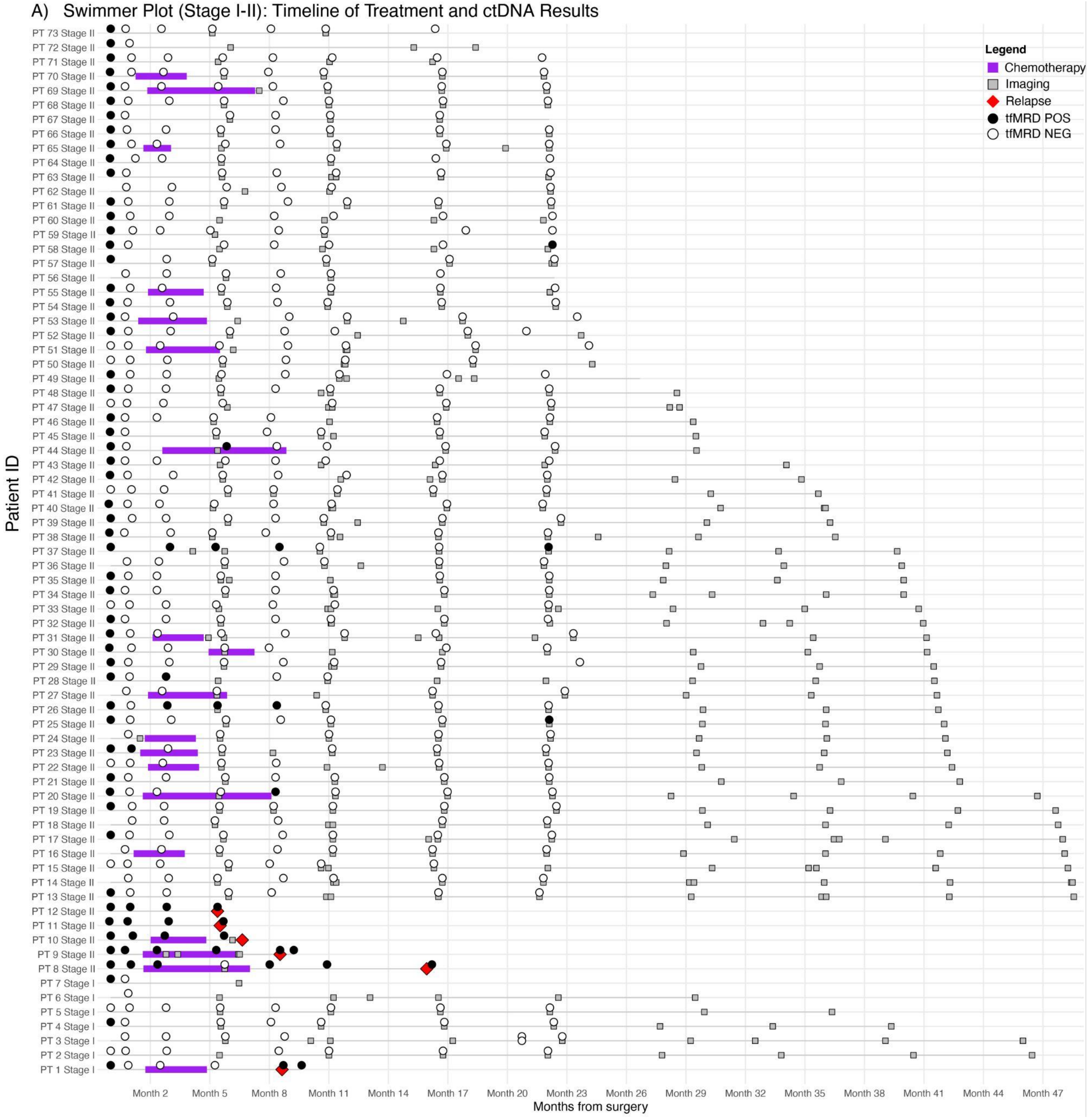

B) Swimmer Plot (Stage III patients): Timeline of Treatment and ctDNA Results

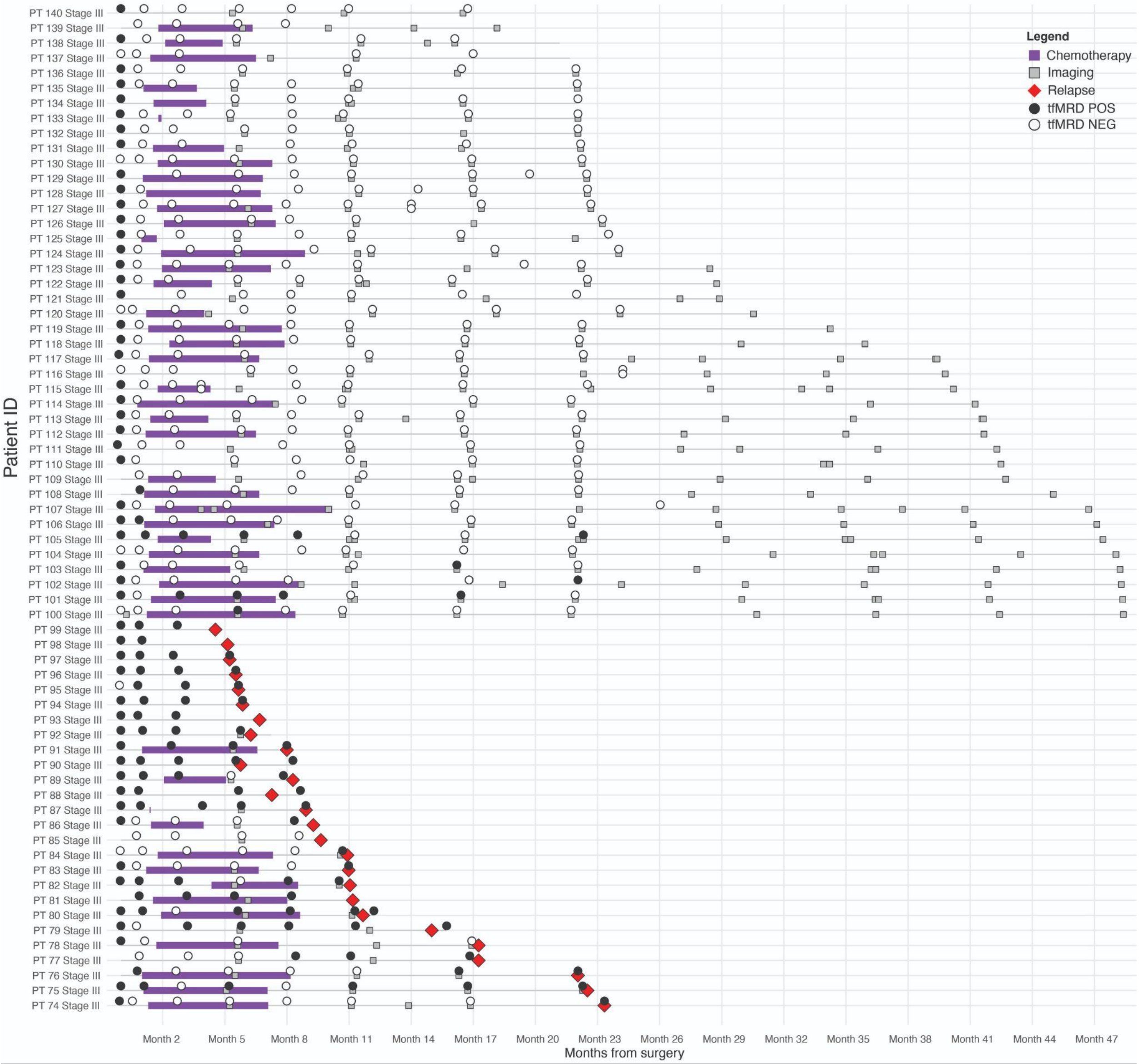

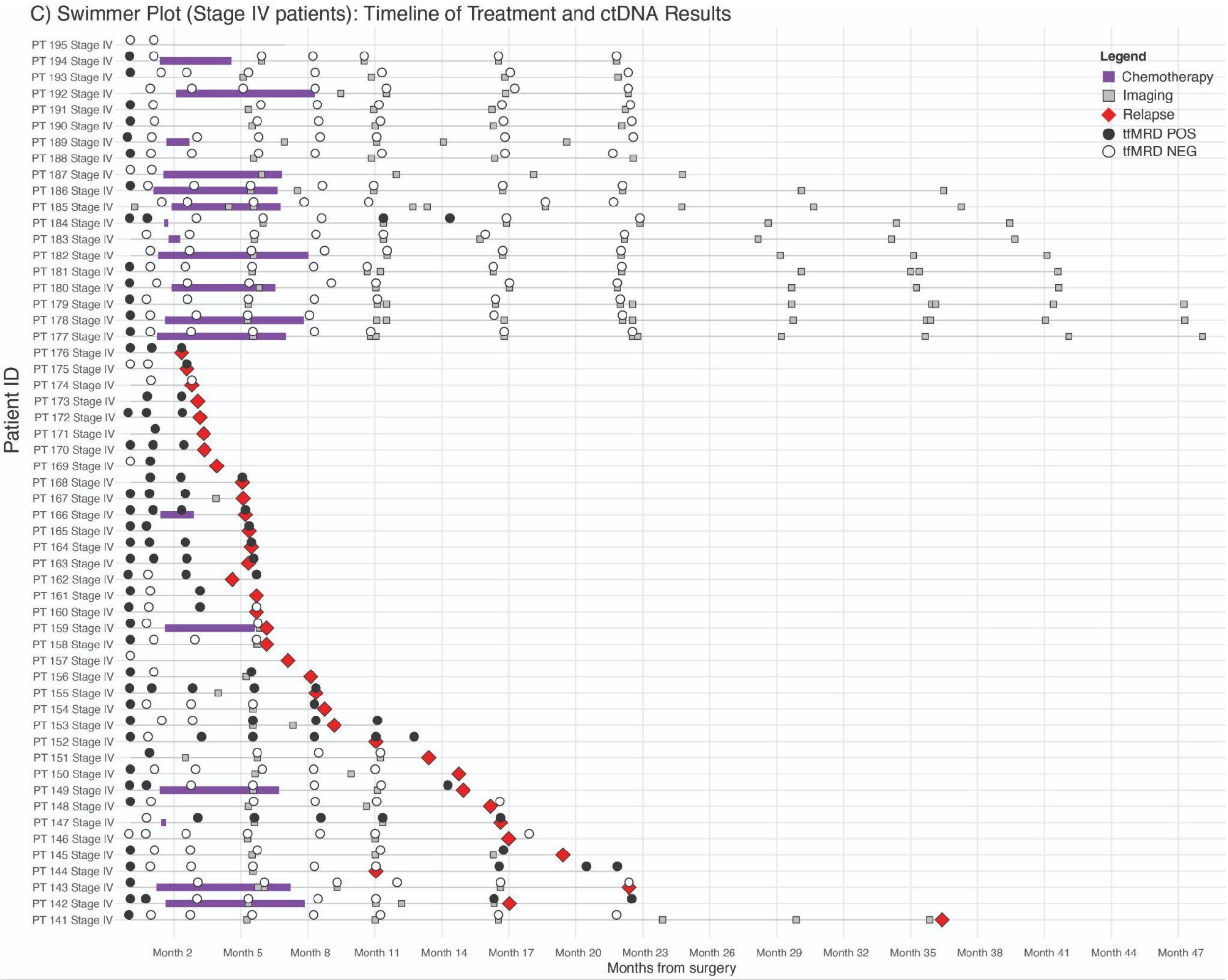

Supplementary Fig 1. Overview plots depicting the complete clinical course of all patients with (A) Stage I-II, (B) Stage III, and (C) Stage IV colorectal cancer, including results of longitudinal ctDNA analysis.
